# A Glance at Aflatoxin Research in Mozambique

**DOI:** 10.3390/ijerph15081673

**Published:** 2018-08-07

**Authors:** Edgar Cambaza, Shigenobu Koseki, Shuso Kawamura

**Affiliations:** 1 Laboratory of Agricultural and Food Process Engineering, Graduate School of Agriculture, Hokkaido University, Sapporo 060-0808, Japan; koseki@bpe.agr.hokudai.ac.jp (S.K.); shuso@bpe.agr.hokudai.ac.jp (S.K.); 2 Department of Biological Sciences, Faculty of Sciences, Eduardo Mondlane University, Av. Julius Nyerere, nr. 3453 Maputo, Moçambique

**Keywords:** aflatoxin, research, overview, Mozambique

## Abstract

In Mozambique, aflatoxin research started in the 1960’s and has been carried through apparently unrelated efforts according to opportunities. However, they can be grouped in two sets: early epidemiological studies and recent agricultural research. Early investigators found a strong correlation between aflatoxin contamination and primary liver cancer. Since then, there have been efforts to examine the extent of contamination, especially in groundnuts and maize. More recent investigations and interventions aimed mostly to reduce the level of contamination, enough to allow such commodities to gain acceptance in the international market. The current status of knowledge is still marginal but the increasing involvement of local authorities, academia, and international organizations seems promising.

## 1. Introduction

Aflatoxins are undoubtedly the most studied mycotoxins in Mozambique, especially aflatoxin B_1_ (AFB_1_). This is due to records of local epidemiological associations between this toxin and hepatic cancer [1], and export restrictions of contaminated groundnuts and maize [2]. There are reports on fumonisins and other toxins [3,4], but these are few compared to the information on AFB_1_. Yet, there is still a need for surveys on food safety covering several foods in the entire country.

The paper by Van Rensburg, et al. [1] on hepatocellular carcinoma (HCC) and AFB_1_ consumption is certainly the most cited about this toxin in Mozambique and one of the most well-known about the etiology of liver cancer. It was an update of Van Rensburg, et al. [5] and Harington, et al. [6] about cancer in Southern Africa. However, these reports were issued over 30 years ago and might be outdated to some extent. Since then, Mozambique has undergone major socio-demographic and political changes. Also, they only covered a few areas in the south of Mozambique. Since then, there have been updates reflecting new challenges in health sciences and agriculture. 

This article provides an overview of the Mozambican aflatoxin research, covering its historical aspects, features, main findings and potential or actual implications for health and society. Such information will be useful for scholars and researchers, students, professionals, trade partners and decision-makers who have a stake in the country’s development.

## 2. Research Driving Forces 

This historical review is based on events’ interconnections rather than a straight timeline exposing them. The time was also considered and there is a notable distinction between the early and recent eras, from a practical perspective, to be explored in this document. Yet, there is a rationale behind this distinction. One cannot detach the events from the sociopolitical context because it defines the country’s priorities and actions.

The initial studies were performed during the pre-democratic Mozambique era, mostly taking an epidemiological approach. These date back to the colonial era (before 1975) and most of its legacy, even during the civil war (until 1992) and adaptation to the new democratic regime (roughly the entire 1990’s). South African gold miners initially supported these studies as an effort to be aware of the health of their potential and actual workers and their families. It explains why most studies were performed in the southern Mozambican provinces. The post-independence government of Mozambique did not support apartheid and it certainly affected the collaborative work between both countries. Any South African scholar or intellectual circulating in Mozambican territory would be seen as suspicious. In 1994, when the African National Congress rose to power, the regime changed and it affected the industrial policies [7], certainly leading it to new directions. Furthermore, most people leading this line of research must have aged and retired. This line of research still exists but it is not so active without the initial incentive.

The most recent studies can be seen as the ones from this millennium, and they are more focused on agriculture. The entire global landscape was influenced by the Millennium Development Goals (MDG) set by the United Nations in 2000 to support developing countries [8]. The goals encouraged the country to design a National Plan to Reduce Poverty [9]. The first MDG was to eradicate extreme poverty and hunger. It clearly encouraged efforts from food supply chains, especially the primary sector (agriculture and fishery). Fields such as food safety, nutrition, and quality gained unprecedented attention, though food security was the main focus. Some millennium goals were towards health but infectious diseases and anemia have more priority than cancer in Mozambique. The deadline for the goals was 2015 and there are now the Sustainable Development Goals [10]; however, the direction seems to be the same. 

## 3. Overview in Different Commodities

**Figure 1:**
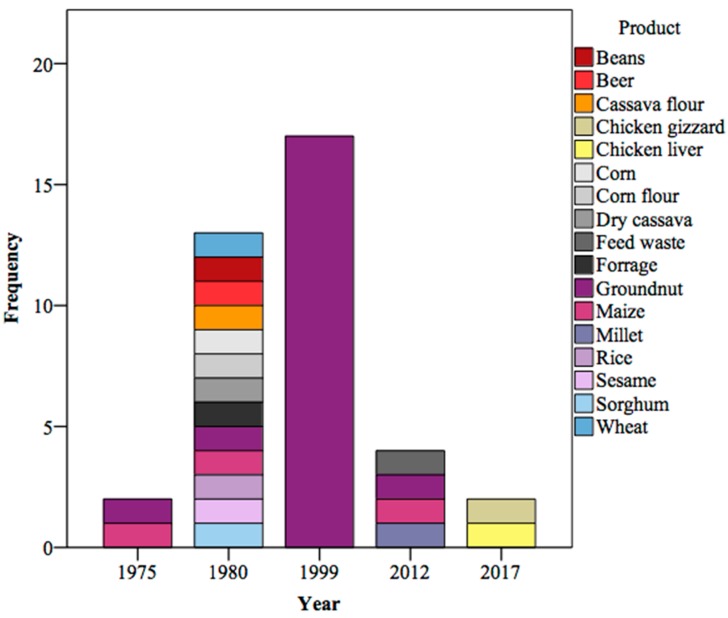
Volume of major aflatoxin research in Mozambique considering the variety of products and frequency of samples. Based on Casadei [11], Van Rensburg, et al. [1], van Wyk, et al. [12], Warth, et al. [4] and Sineque, et al. [13]. These studies were carried from 1985 to 2017 and included in total 934 samples including agricultural crops, chicken livers and gizzards.

Figure 1 shows a meta-analysis on the variation in products and how many samples were analyzed and the results published in Mozambique since the beginning. Some studies were excluded for lack of complete data or consistent information but the most relevant were included.

Groundnuts have always been “on the spotlight”, followed by maize. These were the first crops analyzed and the results were published at the time of the independence. Five years later, there was an inclusion of several products, probably to know which other deserved attention. In the following two decades, the focus was exclusively turned to groundnuts, in an unprecedented level of research. Recently, other commodities have been included. These include feed and animal products.

Table 1 shows in more detail the findings form the last decade. There are very few records, all originating from two studies. One used chromatography plus spectroscopy to analyze plant samples from the Nampula Province and the other used immunoassay to analyze chicken giblets in Maputo City.

Feed waste showed the highest AFB_1_ level, but it does not pose a risk for health as it is not for human or animal consumption. Furthermore, the feed waste and millet samples cannot be considered as representative (*n* < 8). Maize seems to be the most heavily contaminated food, perhaps because most effort to prevent aflatoxin contamination is turned to groundnut. Chicken giblets showed considerable prevalence but the toxin levels were safe for human consumption below the limit recommended by Codex Alimentarius (10 µg/kg) [15].

## 4. Early Studies: Biomedical Research

Aflatoxins shall be assumed as a burden since pre-historic times and as a public health issue since the dawn of civilization. Mushroom and mold poisoning are not exactly a novelty. However, many authors regard the turkey X disease episode [16] as the kick-start of mycotoxin research. According to Homei and Worboys [17], Brazilian feed resulted in the death of over 100.000 turkeys in England. It turned out to be a result from contamination by AFB_1_ [18]. The fear of such kind of incidents originated a worldwide cascade of intense research as well as policymaking to ensure food and feed safety. Now mycotoxin studies are a well-established multidisciplinary body of knowledge combining contributions from medical, agricultural, legal, and other disciplines.

An early record of aflatoxins in Mozambique was issued by Mota and Lourenço [19] in Agronomia Moçambicana. They found the AFB_1_ in cassava in levels exceeding the limits admissible for human consumption. However, it was only an isolated study focused on the nutritional and microbiological features, rather than toxicological. Thus, there was little following in such direction. One worth mentioning is Essers and Nout [20] who still believed samples were contaminated, even after failing to detect them. They based their conclusion on previous records, a flaw in their method previously explained by Coker and Tomlins [21], and probably their impression by witnessing the level of mold infection. Yet, there was not much interest in aflatoxins because they were actually looking for cyanogenic glycosides. Aflatoxin analysis in cassava seems marginal, performed just because opportunities rise, perhaps because the most recent attempts showed no evidence of contamination [20]. 

Groopman, et al. [22] mentioned studies on cancers and their etiologies for the period 1968–1974 in Inhambane. This was certainly the first time interest in aflatoxins has truly risen in Mozambique. Discoveries by Purchase and Goncalves [23] followed over the next two decades of research. They found aflatoxins in food from cancer patients and mentioned groundnuts as a major source. Van Rensburg, et al. [5] gave continuity and their observations were presented in the following year as strong epidemiological evidence of an association between HCC and aflatoxins in the a Conference on Mycotoxins in Human and Animal Health [24]. At that time, Kew, et al. [25] described HCC as the most frequent cancer in the south of Mozambique. In 1979–1980, the National Institute for Agricultural Research (INIA, now IIAM) surveyed aflatoxins in 17 commodities [11,26]. Most samples were contaminated, especially the grains. Later, Van Rensburg, et al. [1] issued a very influential paper on aflatoxins, demonstrating their combined impact with hepatitis B virus (HBV) in the onset of HCC. Their epidemiological study demonstrated that liver cancer was more frequent among HBV-positive individuals who had high levels of aflatoxin B in their diets.

Later, dietary aflatoxin intake was found to be associated with a codon 249 mutation of the p53 gene in an endemic form of HCC [27]. However, Unsal, et al. [28] demonstrated that HBV also has an important role in the wild-type p53 function, and this mutation seems rare outside Mozambique, even in other places with a high incidence of HCC and aflatoxin contaminated food. Sarmento, et al. [29] carried out a pertinent following in this line of research, analyzing microsatellite instability in HCC among Portuguese and Mozambican subjects. However, their focus was only cancer, not exploring in depth the relationship between it and aflatoxins, HBV, or any other possible causes. Yet, they mentioned the toxins among the most probable causes of the differences between both populations analyzed.

These studies were very enlightening and they have a lot of potential in three major directions: the aflatoxins in food, hepatitis, and liver cancer. Each can be developed into a comprehensive research topic in Mozambique. Even the work already done can simply be updated and reproduced in different areas. New molecular and immunological approaches, and information technologies can be a valuable asset.

Two remarks before moving forward: beware of biases because of the timeframe and misleading secondary sources.

It is important to consider the timeframe because the mycotoxin research and policymaking outside Mozambique have advanced substantially. For example, investigators such as Mota and Lourenço [19] describe their samples as unacceptable for human consumption, but that seems subjective or even arbitrary by today’s standards. By that time, there was no national legislation or international agreement on what should be considered acceptable. The International Agency for Research on Cancer (IARC) [30] only published its evaluation of aflatoxins over two decades later, and the Codex Alimentarius Commission [15] issued its Standard for Contaminants and Toxins much later. Yet, these opinions on the acceptability can be accounted as professional subjective observations.

## 5. Recent Studies: Trade Affairs

Since 1999, the focus of AFB_1_ research shifted from studies on hepatic carcinoma to agricultural concerns. This was due to international trade restrictions, especially regarding groundnuts [2,31]. A study by van Wyk, et al. [12] pioneers this direction. According to them, it all started when European countries started to block the entrance of AFB_1_ contaminated groundnuts from South African companies supplied by farmers from Nampula province in Mozambique. When they investigated products, they found AFB_1_ levels extremely high.

The 2004 major aflatoxicosis outbreak in Kenya [32] also explains the increased concern about AFB_1_. There had been similar incidents in India [33], in 1975, and Malaysia [34], in 1991, but the one in Kenya drew more overall attention and media coverage, probably because of today’s efficient information technologies and the higher number of confirmed deaths, estimated to more than 125 by the Centers for Disease Control and Prevention [35]. After this, a group of experts reunited in the World Health Organization’s headquarters to develop public health strategies to prevent or control future outbreaks [36]. This is a major concern to Mozambique because both countries have AFB_1_, a major grain contaminant, and share several natural and socio-demographic features.

Mondlane, et al. [37] started by screening for *Aspergillus* infection and AFB_1_ contamination and analyzed feed for poultry, using samples from four companies. It represented an ensemble effort from the University Eduardo Mondlane, Ministry of Health and the Mozambican Institute for Agricultural Research. Most samples had *A. flavus* and showed aflatoxin levels above the limits recommended by the Codex Alimentarius. The infected samples had significantly higher toxin levels (*p* = 0.003), though some non-infested samples also had AFB_1_ in minor quantity. It is reasonable, as the industrial treatment probably eliminated partially the molds but not the toxin. Indeed, Carlson and Ensley [38] do not recommend fungal infection as a predictor of mycotoxin contamination. Nevertheless, the research was an asset for the companies and farmers in general.

A remarkable development in the following years was the introduction of Aflasafe^®^ a bio control formulation based on atoxigenic *A. flavus* strains [39]. It is used to reduce aflatoxin production by *Aspergillus* [40,41]. The International Institute of Tropical Agriculture (IITA) developed this product and brought it to Mozambique in collaboration with Eduardo Mondlane University and Lúrio University, and the financial support of the United States Department of Agriculture (USDA) [41]. Cardwell, et al. [42] claim this product to be responsible for the aflatoxin reduction in maize and groundnut by 80 to 90% in Nigeria. They say it was also successful in Kenya. It is now sold in Nampula province of Mozambique, where IITA has an office.

Augusto, et al. [2] studied the prevalence and distribution of *Aspergillus* in soil samples, and aflatoxin contamination in maize and groundnuts. It covered areas from Nampula, Zambézia, Tete, and Manica provinces. The first two showed more abundance of molds and the highest levels of contamination. This study confirmed Nampula as an “aflatoxin hot spot” area, AFB_1_ in Mozambique major cash crops, and expanded the studies to three other provinces. Furthermore, it raised awareness about the high levels in Zambézia. Two years later, Zuza, et al. [43] published another study of AFB_1_ in groundnuts from Nampula. However, this group wanted to find out how the harvesting time affected the toxin levels. According to them, the farmers have to harvest at the exact time of physiological maturity. They believe a delay results in the invasion of the pods by insects and the holes allow the mold’s entry. This recommendation was essential, not only for the safety but also for the groundnut’s quality.

At the same time, Anjos, et al. [44] were also concerned about the exposure, and how to reduce it. However, they explored a completely different perspective and context: there had been studies on how adsorbents can reduce the toxic effects of aflatoxins [45,46]. Thus, they focused on bentonite clay and diatomaceous earth, and tried to see how they would affect the health of chicks. Only bentonite clay showed efficacy, though only moderately. This result was promising because the adsorbent is abundant in Mozambique.

Sineque, Macuamule and Dos Anjos [13] also drew their attention to poultry, screening chicken livers and gizzards for AFB1. The samples were from small and industrial abattoirs of Maputo. There were more aflatoxins in livers than gizzards, and the values were not above the limits recommended by the Codex Alimentarius. There is no apparent reason why a gizzard should have a high aflatoxin level, as the food only passes through. Concerning the liver, one of its functions is to destroy hazardous chemicals [47], so it naturally reduces the quantity of such substances. Yet, it is the organ where these substances accumulate for the same cause. Thus, any amount of toxin should be expected from chicken after eating contaminated feed. The initiative was good and unique for exploring animal food, and relevant as liver and gizzard are widely eaten as a delicacy in Mozambique. 

It is also important to admit the existence of a wide unpublished body of work, made for particular consultancy purposes. It is quite frequent in Mozambique. Furthermore, many students make resourceful work as part of their education but the information is archived once they finish, and it never “sees the light of day”, and many laboratories performed analyses by demand or routine but the results are never explored academically. Thus, there are huge volumes of unexplored databases in Mozambique. They could bring answers and certainly fill some gaps in the current knowledge on the country’s aflatoxin research.

These studies certainly give some insight on the country’s current situation, but much more is required to draw the “full picture”. All authors mention the need for further investigations because their studies are too limited to be conclusive. Each had unique features, probably depending on opportunities and intentions, but the combined contribution show how much is known about AFB_1_ in Mozambique and the direction to follow. The research is marginal if compared with the body of material produced in other regions, but most studies here mentioned are pioneers in their own right. The next generation of researchers shall see this as an opportunity to lead in this matter.

## 6. Perspectives

There have been several efforts worldwide to control aflatoxin contamination, all trying to tackle one or another aspect of the issue. Some scientists are focused on the toxin’s epidemiology [14,48,49,50], pathology [51], risk management [52,53], some on intervention or legal issues [54,55,56,57], and several technical reports show improvements in some detection methods [58,59,60] or biological control [61,62,63,64]. There seems also to be an increasing trend on aflatoxin M_1_ (AFM_1_) [65,66], though it still seems “underrated” given its relevance for infant and general public health. Food detoxification is another direction under consideration [67], although it is still difficult to eliminate aflatoxins without compromising the quality of the food [54]. These studies might provide useful clues on which direction to follow.

It is important to be aware of the current developments on the tools for aflatoxin analysis because the future of will highly rely on their quality [68]. Scientists have been developing highly sensitive tools for AFB_1_, many of which are described in their extensive reviews [60,69,70]. Liu, et al. [58] developed a simple and stable immunosensor based on CdS–Fe3O4 magnetic nanocomposites capable of detect the toxin at 5 pg/mL. Prihantoro, et al. [71] recently purified an antibody to be used as a sensor for AFB_1_, but there is still a need to identify it.

Some studies have been more focused on how to predict contamination and manage the risk. Battilani [53] published a brief article on the matter, stating that they will gain relevance over time because of climate change. Mycotoxin contamination highly depends on environmental factors such as temperature and water activity [72], and they are expected to change considerably in the future. Navarro, et al. [52] developed an online application based on geographical information systems (GIS) to assess aflatoxin risk in Georgia, United States. It is worth mentioning a growth study of *Fusarium graminearum* by Cambaza, et al. [73] demonstrating that mold surface color is a potential tool to analyze the fungal state of maturity, possibly to estimate the amount of toxin produced by the fungus. This technique can later be used to monitor aflatoxin synthesis by *Aspergillus*. The method is not costly and can be applied with resources already available in Mozambique [54].

Molecular and genomic studies are perhaps among the most promising approaches in aflatoxin research, though there is still a considerable need for improvements [74]. According to Bhatnagar, et al. [63], there is a rising predominance of three major directions: biological control, through atoxigenic *Aspergillus*, identification of resistance factor in maize, and bioengineering of susceptible crops to improve resistance. Some molecular studies have been prominent. For instance, Thakare, et al. [61] developed an aflatoxin-free transgenic maize using host-induced gene silencing, and Abbas, et al. [62] conducted research on the biological control of aflatoxin contamination in U.S. crops and the use of bioplastic formulations of *A. flavus* biocontrol strains to optimize application strategies. These studies seemed very advanced and costly, perhaps currently impractical to be conducted in Mozambique, though the country is currently producing and selling Aflasafe [40].

A recent review by Ismail, et al. [67] mentions several decontamination methods under experimentation, some are able to keep food safety, quality and nutritional value to acceptable levels. They mentioned gamma radiation and ozone for some foods, and also bacterial or yeast degradation of the toxin. But they said there is still a need to clarify the mechanisms of detoxification and how it can affect organoleptic properties of foods. The study by dos Anjos, et al. [44] falls into this category, as they were trying to reduce the toxic effect of contaminated feed by using adsorbents. Indeed, their results were promising but there was no follow up. It shows Mozambique’s potential to pursue this direction.

There are efforts to clarify the mechanism of AFB_1_ toxicity and how to cure or reduce it in the liver. For example, Krishnappa, et al. [51] tested the effect of garlic extract in the liver of Winstar rats damaged by aflatoxin B_1_ and their results were satisfactory. Yet, this line of research is not likely to become a priority in Mozambique, though some key studies in the area were conducted in the country or based on the Mozambican population [1,25,27,29]. Among the reasons, the following can be considered: there is already some information on the matter in the country and much more locally known, though not available in the mainstream international literature. Many other countries have resources to better conduct such kinds of approaches. Agricultural and trade matters are now the priorities, therefore, most studies will likely be related to these fields. Even more feasible are studies on how good agricultural practices (GAP) can minimize aflatoxin contamination. Some major studies on the matter, such as the recently published by Torres, et al. [75] on GAP in peanuts can be a starting point for future researchers in Mozambique.

Researchers unanimously agreed there is a need for further research on aflatoxin contamination in Mozambique. Some recommended studies about the incidence and extent of contamination, some point to specific commodities such as groundnuts, maize, poultry giblets, milk or others, some talk about the methods for research, improvement of the food, or to reduce the exposure, some about the regularity of the analysis, and some do not specify the path to take. In the end, whichever the direction is, any research on aflatoxin is a step forward.

Aflatoxin studies are multidisciplinary, including biomedical, chemical, agricultural, social, legal and several other components. Mozambique has a lot of potential from any of these fields and they will sooner or later be addressed. In areas such as Maputo, Inhambane, specially Nampula and possibly Manica, there will be short to mid-term progress regarding aflatoxin control because they have been drawing attention from researchers and the local authorities. However, the six remaining provinces will probably take much longer unless they keep up with the others.

## 7. Conclusions

Four major forces drove aflatoxin research in Mozambique: cancer studies, international trade demands, scholars’ initiatives, and opportunities. The first two are practical and were necessary at least at some point in time. Researchers will have to rely on grants for cancer studies or and trade-motivated screenings as support and that is what creates a few unrelated events of intense research. The opportunities frequently come from foreign investment or request for commercial purposes, rarely extending beyond that. Some of the best studies do not even involve Mozambican scholars, professionals, or institutions. Furthermore, most papers on aflatoxins in Mozambique are either for conferences or one-time studies rather than part of control campaigns, though recently the overall interest in food safety has been increasing.

Mozambique is among the countries most affected by AFB_1_, in health and economy, and this should be a cause for the country to lead in this line of research. There are currently more facilities than ever before, though screening studies require sophisticated and onerous material. Furthermore, there are other aflatoxin related issues to study, which are probably more feasible yet not as relevant. Why not, for example, try to search for ways to control for known aflatoxigenic *Aspergillus* strains? Or, analyze its growth pattern or how environmental factors affect mold? What other crops than groundnuts, maize, and cassava are being affected? Why not to perform studies on economic losses? There is no need for much money in order to expand the knowledge about aflatoxin in Mozambique. Different sectors could try to study the best way to deal with this issue.

Aflatoxin research requires two more forces to truly flourish: general public concern and the local market’s interest. It is necessary to create awareness through mainstream education and media. Aflatoxin-related disorders should be as known as other food borne diseases. They are endemic in Mozambique and they will not decrease in prevalence unless there is some intervention. Regarding the market, Mozambique will be much better prepared to meet the global requirements if the local policies are also demanding. It might be difficult to implement in the beginning but high quality products will also encourage transnational partners to purchase more from Mozambique. The local traders will keep selling low quality products if the clients do not mind buying them as they are. And clients will never change their behavior without awareness.

One shall praise the effort so far in the Mozambican aflatoxin research and the achievements. The researchers have done much regarding their very limited resources. Yet, there are many challenges yet to come, especially regarding the way the different actors will have to participate in the control of aflatoxins. Above all, it will be almost impossible to do anything if the aflatoxin research does not advance substantially.

## Figures and Tables

**Table 1:** Aflatoxin levels detected in Mozambican products since 2012. Based on Cambaza, et al. [14].

Year	Analytical Method	Product	Sample Size (*n*)	Prevalence (%)	Average (µg/kg)	Median (µg/kg)
2012	LC-MS/MS *	Feed waste	1			433.0
		Groundnut	23			3.4
		Maize	13			69.9
		Millet	2			4.0
2017	ELISA **	Chicken gizzard	80	13.8	1.1	
		Chicken liver	100	39.0	1.7	

* Liquid chromatography tandem-mass spectrometry, ** Enzyme-Linked Immunosorbent Assay.
